# Peer Group Embeddedness and Academic Motivation: A Developmental Perspective

**DOI:** 10.3389/fpsyg.2021.701600

**Published:** 2021-09-09

**Authors:** Marion Reindl

**Affiliations:** Educational Science, Paris Lodron University of Salzburg, Salzburg, Austria

**Keywords:** peer group embeddedness, academic motivation, early adolescence, change models, development

## Abstract

The present study focused on the increasing importance of peer group embeddedness on domain-specific academic motivation (intrinsic value and mastery goals) over the course of early adolescence. In this regard, two important research questions were investigated: (1) Does a change in peer group embeddedness influence a change in student intrinsic value and mastery goals? (2) Does this influence increase over the course of early adolescence? The research questions were investigated based on a five-wave longitudinal study over two school years (seventh and eighth grade) in Germany. The final sample comprised 349 students. True- intraindividual-change models showed a positive effect of a change in peer group embeddedness in the first half of eighth grade on the change of all domain-specific motivational dimensions—except for intrinsic value in English—in the second half of the eighth grade. In the seventh grade, a change in peer group embeddedness had no effect on all motivational dimensions. The results were discussed in terms of taking a developmental perspective for both peer group embeddedness and student academic motivation.

## Introduction

In a rapidly changing technological and globalized world, students are constantly required to expand their knowledge, e.g., in mathematics and English, to meet the upcoming demands in their lives. In this regard, Students’ motivation plays an important role. For example, if students enjoy the learning activity (intrinsic value) and want to improve their knowledge (mastery goals) without focusing only on the next exam, Students’ willingness to learn new skills by themselves might be more likely—even after their school career (Achtenhagen and Lempert, 2000). However, during early adolescence, Students’ intrinsic value and mastery goals change, often in an unfavorable direction (Gottfried et al., 2001). More importantly, it seems to identify conditions under which Students’ motivation may develop positively at this developmental stage.

During early adolescence, peers become important socialization agents (Brown and Larson, 2009). In addition to the dyadic perspective, the group perspective becomes especially important because most Students’ interactions take place in larger peer groups at this developmental stage (Rubin et al., 2007). One important mechanism by which peer groups may influence motivational development is based on Self-determination theory (Deci and Ryan, 2000) and the assumption that the level of peer group embeddedness may promote internal forms of motivation, such as intrinsic value and mastery goals.

The present study contributes to the existing literature by taking a developmental perspective in two ways. First, in addition to academic motivation, peer group embeddedness is assumed to be a dynamic and rapidly changing phenomenon during adolescence. Therefore, developmental trajectories were focused on both peer group embeddedness and academic motivation and investigated whether a change in peer group embeddedness predicted a change in dimensions of academic motivation. Second, due to the increasing importance of peer groups during adolescence (Rubin et al., 2007), changes in the strengths of effects were investigated over 2 years based on a longitudinal study with five measurement occasions. In this regard, two important research questions were investigated: (1). Does a change in peer group embeddedness influence a change in student intrinsic value and mastery goals? Does this influence increase over the course of early adolescence?

### Academic Motivation

The present study focused on two important motivational concepts for learning and achievement, namely, intrinsic value (Wigfield and Eccles, 1992, 2002) and mastery goals (Nicholls, 1984). Intrinsic value is defined as the enjoyment doing a task (Wigfield and Eccles, 1992, 2002), whereas mastery goals focus on learning new skills and developing competence (Elliot and Murayama, 2008). Both concepts have in common the view that the execution of activities is driven more by self-referential personal standards, e.g., to have fun or to learn than by external consequences.

From a developmental perspective, studies provide evidence that intrinsic value (Fredricks and Eccles, 2002; Jacobs et al., 2002; Frenzel et al., 2010) and mastery goals (Scherrer et al., 2020) decline over the course of the secondary school years. With respect to the domain-specific focus on mathematics and English, the magnitude of the change can vary due to Students’ preferences for specific topics (Baumert and Köller, 1998). This is also reflected in significant variations in these developmental trajectories in the domain of mathematics (Frenzel et al., 2010; Reindl et al., 2015; Scherrer et al., 2020) and English (Gottfried et al., 2001; Jacobs et al., 2002). Theoretical concepts explain these variations in the developmental trajectories through conditions in the person, e.g., self-perceptions but also through conditions in the social environment (Ames, 1992; Eccles, 2004), such as peer group embeddedness.

### Peer Group Embeddedness: A Social Network Perspective

The peer group can be defined as a “collection of interacting individuals” (Rubin et al., 2007, p. 17) and becomes especially important during early adolescence because most Students’ interactions take place in larger peer groups at this developmental stage (Rubin et al., 2007). One important group constellation in adolescence is the clique. This is a self-selected and friendship-based group, whereas not all members have to be connected through a reciprocated friendship (Hinde, 1976; Rubin et al., 2007).

Peer group embeddedness is indicated by social network centrality (Bonacich, 1987), which focuses on Students’ level of involvement and interactions in their specific clique (Wasserman and Faust, 1994). The index is based on the out-degrees (nominated students) and in-degrees (nominations of students) weighted by the relative connections of the persons in the network. Thus, the level of Students’ embeddedness is dependent on the number of friends and the connections that those friends have (Andrews, 2020).

Taking a developmental perspective, the results of a meta-analysis from Jiang and Cillessen (2005) showed that during adolescence, familiar facets of peer group embeddedness, e.g., Students’ acceptance, changed to a considerable degree, reflected by an average autocorrelation of *r* = 0.50. This phenomenon could be explained by the fact that students change their friendship networks relatively often during early adolescence (Meter and Card, 2016). The peer group represents an important reference group for comparing and validating values and in turn increasing security of identity. If peer group-specific norms and values do not fit with those of the students, separation from the group—a decision from the person or/and the group—and seeking a new one might be more likely.

### Peer Group Embeddedness and Academic Motivation

Explanations for the influence of peer group embeddedness on motivation can be derived from Self-determination theory (Deci and Ryan, 2000) in combination with support concepts (Ladd et al., 2009). With respect to Self-determination theory, peer group embeddedness is closely related to the basic need for relatedness, indicating that the more students are involved in their specific clique, the greater the need for relatedness is fulfilled. The feeling of being connected to other students may cause positive emotions, allowing students to deal with subject-specific tasks more enthusiastically. Additionally, highly connected peer groups may provide a more supportive—and less competitive—environment (Mundt et al., 2017). Thus, highly embedded students receive more emotional and instrumental support from their peers than less embedded students. Positive emotions in combination with a supportive environment should provide the basis that highly embedded students experience more enjoyment and want to improve their knowledge without outperforming others. In contrast, when students are weakly connected in a group with other students, i.e., Feeling rejected by other group members, experiencing positive emotions and having a supportive environment should be less likely. Those students should get faster board and worried (Furrer and Skinner, 2003) and, in turn, more in risk for an unfavorable development of their academic motivation.

Most of the studies that focused on the relations between peer group embeddedness—or familiar concepts—and motivational outcomes are on a correlational level. A meta-analysis by Wentzel et al. (2020) showed low but stable relations between acceptance and academic motivation (negative affect or active engagement). Regarding effects on the change of academic motivation, a study from Wang and Eccles (2012) showed that students who feel supported by their peers had lower declines in their academic motivation during adolescence compared to students who feel less supported. Focusing on a change in both peer group embeddedness and academic motivation, only one study was found. De Laet et al. (2015) conducted a longitudinal study with elementary school children over 3 years. The results provide evidence that peer acceptance in the fourth grade predicted a change in behavioral engagement over three school years. In contrast, the change in peer acceptance over three school years was not related to the change in student behavioral engagement.

### Increasing Influence in Early Adolescence

Theoretical explanations for the increasing importance of peer group embeddedness for student academic motivation can be derived from Individuation theory (Youniss and Smollar, 1985) adapted to the academic context. Individuation theory acknowledged that students want to be part of a group to distance themselves from their parents. In this regard, peer connections might fill a gap before students become independent from their parents as autonomous persons (Youniss and Smollar, 1985; see also Steinberg and Monahan, 2007). With respect to the academic context, with increasing grade levels, parents may be less able to support their children on school content. Thus, peers at the same grade level—working on the same school content—may gradually take the parents place in supporting students emotionally as well as instrumentally to overcome motivational dilemmas. Empirical evidence about the increasing importance of peer group embeddedness on motivation during early adolescence is rare. Studies mostly combine different age groups or investigate developmental stages such as childhood and adolescence (e.g., Wentzel et al., 2020).

### The Present Study

Based on Self-determination theory (Deci and Ryan, 2000) and support concepts (Ladd et al., 2009) embedded in a developmental framework (Youniss and Smollar, 1985), the present study focused on two important research questions: (1) Does a change in peer group embeddedness influence a change in student intrinsic value and mastery goals? (2) Does this influence increase over the course of early adolescence?

Nearly all of the reported studies related to the first research question and focused on motivational outcomes are on a correlational level. Even studies that implemented a longitudinal design used mostly cross-lagged analysis focusing only on changes in the rank order between measurement occasions (Rogosa, 1995). Very few studies have analyzed intraindividual changes in the development of motivation. Therefore, most of the aforementioned studies provide evidence for the relations between peer group variables and academic motivation. This is an important first step to investigate whether peer group embeddedness—or related concepts—and motivation are associated. An important next step to capture the socialization influences of peer groups during early adolescence is identifying how they create changes in the development of motivation (Kindermann, 2016). Therefore, this study focused on intraindividual changes in internalized forms of academic motivation in mathematics and English and how they were influenced through peer group embeddedness. Moreover, although studies provide evidence that peer group constellations are a dynamic and malleable phenomenon, only one study focused on the change in a familiar concept of peer group embeddedness, e.g., acceptance, and how the change is associated with academic outcomes. This study took a long-term perspective over 3 years and found no relations. However, as Students’ peer relationships also change to a considerable degree within a school year, it is important to investigate how such short-term changes may influence student motivational development. Therefore, the hypothesis was formulated as follows:

H1: The more students become embedded in their peer group, the more favorable the development of their intrinsic value and mastery goals (mathematics and English).

With respect to the second research question, although theoretical concepts acknowledged an increasing influence of peers during early adolescence, most of the studies combined different age groups, neglecting to investigate how the influence of peer group embeddedness increases over the course of early adolescence. Therefore, the hypothesis was formulated as follows:

H2: The influence of peer group embeddedness on intrinsic value and mastery goals (mathematics and English) increases during early adolescence.

The present study goes behind the existing literature in several regards. First, both peer group embeddedness and dimensions of academic motivation were seen as rapidly changing phenomena during early adolescence. Second, two important subjects, mathematics and English, were focused on validating the results across different subjects. Third, the conceptualization of the present study allows us to focus on peer group influences at different developmental stages in early adolescence.

## Materials and Methods

### Participants and Procedure

The sample is from a larger German longitudinal study that focused on the influence of peers on academic motivation. Students was followed over two school years to capture the increasing importance of peer influences in early adolescence. The measurement occasions were scheduled in the beginning of the seventh grade (time point 1), in the middle of the seventh grade (time point 2), in the beginning of the eighth grade (time point 3), in the middle of the eighth grade (time point 4), and at the end of the eighth grade (time point 5). The Ministry of Education of the Federal State of Baden-Württemberg approved and supported the study. Students were recruited through the school they attended. Informed consent for participating in the study was given by the principals of the schools, the teachers, and the parents. The data collection was conducted by trained research assistants.

In sum, 397 students took part at the first measurement occasion. As developmental trajectories in peer group embeddedness and academic motivation were focused, only students who participated in at least two measurement occasions were chosen. Therefore, students who participated in the first measurement occasion were excluded (*n* = 48). The final sample consisted of 349 students. Several *t*-tests were calculated comparing the variables of interest between students who dropped out and students who remained at the study. The results revealed that students who dropped out did not differ regarding their peer group embeddedness, *T*(394) = −0.11, *p* = 0.912, their intrinsic value in both subjects, *T*(395) ≤ |1.81|, *p* ≥ 0.071, and their mastery goals in both subjects, *T*(395/55.95) ≤ |1.97|, *p* ≥ 0.053. The final sample consisted of 180 male students and 169 female students from 19 classrooms. A total of 163 students attended a higher school track, and 185 students attended a lower school track. The socioeconomic status of the participating families ranges from 14.3 to 88.70 (ISEI, International Socio-Economic Index of Occupational Status).

### Measures

#### Intrinsic Motivation and Mastery Goals

Students reported on their intrinsic value on a standardized German questionnaire (Steinmayr and Spinath, 2010). The three-item scale assessed to what extent students enjoy the respective subjects (“Mathematics/English is fun to me”). Mastery goals were assessed by an adapted shortened version of a standardized German questionnaire (Spinath et al., 2002). The scale assessed with four items to what extent students want to learn new skills and develop competence in the respective subject (“In Mathematics/English I want to learn something interesting”). All items were rated on a five-point scale, ranging from 1 (*completely not true*) to 5 (*completely true).* The construct reliabilities across the domains mathematics and English for all time points are very good (see Tables 1, 2). Because this study was part of a larger study on motivation and peers, we applied a multimatrix design (Jorgensen et al., 2014) with three test booklets in which a balanced approach over time was used. Test booklets were randomly distributed within each classroom. This method provides results similar to those obtained with complete datasets (Smits and Vorst, 2007).

**TABLE 1:** Summary statistics for intrinsic value Mathematics and English (latent level).

		1	2	3	4	5	6	7	8	9	10
1	IV T1	(0.93/0.92)	0.73**	0.72**	0.62**	0.61**	0.05	–0.03	–0.03	0.02	–0.04
2	IV T2	0.77**	(0.93/0.92)	0.85**	0.76**	0.67**	0.04	–0.03	–0.02	0.02	–0.02
3	IV T3	0.75**	0.85**	(0.92/0.92)	0.77**	0.80**	0.03	–0.06	0.00	0.05	–0.02
4	IV T4	0.69**	0.79**	0.84**	(0.94/0.91)	0.85**	0.02	–0.04	0.01	0.05	–0.05
5	IV T5	0.65**	0.69**	0.78**	0.84**	(0.92/0.90)	0.04	–0.07	0.05	0.11	–0.04
6	Emb T1	–0.05	–0.01	–0.03	–0.02	–0.02	(/)	0.33*	0.50**	0.40**	0.18
7	Emb T2	0.09	0.07	0.06	0.06	0.10	0.33*	(/)	0.23	0.21	0.28*
8	Emb T3	0.05	0.05	0.07	0.08	0.06	0.50**	0.23	(/)	0.60**	0.20
9	Emb T4	–0.05	–0.06	–0.03	–0.01	0.04	0.40**	0.21	0.60**	(/)	0.36**
10	Emb T5	–0.03	–0.01	–0.01	–0.03	0.01	0.18	0.28*	0.20	0.36**	(/)

*IV, Intrinsic value; Emb, Embeddedness; the values below the diagonal represent the correlations in mathematics; the values above the diagonal represent the correlations in English; the values in the diagonal represent the construct reliability (Jöreskog’s rho) for mathematics/English.*

*p < 0.05, **p < 0.01.

**TABLE 2:** Summary statistics for mastery goals Mathematics and English (latent level).

		1	2	3	4	5	6	7	8	9	10
1	MG T1	(0.77/0.85)	0.80**	0.71**	0.52**	0.53**	0.00	–0.01	−0.09*	–0.02	–0.04
2	MG T2	0.84**	(0.84/0.88)	0.81**	0.72**	0.62**	0.02	0.01	–0.06	0.02	–0.02
3	MG T3	0.83**	0.86**	(0.83/0.87)	0.81**	0.70**	0.04	–0.06	–0.03	0.02	–0.04
4	MG T4	0.76**	0.75**	0.90**	(0.86/0.86)	0.80**	0.00	–0.08	–0.02	0.04	–0.06
5	MG T5	0.65**	0.57**	0.76**	0.80**	(0.85/0.85)	–0.02	–0.09	–0.02	0.09	–0.05
6	Emb T1	–0.03	0.01	–0.02	–0.04	–0.06	(/)	0.33*	0.50**	0.40**	0.18
7	Emb T2	0.03	0.01	–0.01	–0.01	0.03	0.33*	(/)	0.23	0.21	0.28*
8	Emb T3	–0.03	–0.05	–0.02	0.01	–0.07	0.50**	0.23	(/)	0.60**	0.20
9	Emb T4	–0.06	–0.07	–0.05	–0.02	–0.01	0.40**	0.21	0.60**	(/)	0.36**
10	Emb T5	–0.07	–0.01	–0.05	–0.04	–0.05	0.18	0.28*	0.20	0.36**	(/)

*MG, Matery goals; Emb, Embeddedness; the values below the diagonal represent the correlations in mathematics; the values above the diagonal represent the correlations in English; the values in the diagonal represent the construct reliability (Jöreskog’s rho) for mathematics/English.*

*p < 0.05, **p < 0.01.

#### Peer Group Embeddedness

For the assessment of peer group embeddedness, the centrality measure of Bonacich (1987) was used. A precondition for the calculation of network centrality is that Students’ cliques had to be identified. Therefore, unlimited peer nominations were used as recommended for the assessment of group constellations (Gommans and Cillessen, 2014). Adolescents were asked to list students “who hang around with” in a ranked order.

#### Control Variables

Teacher-reported grades of the last exams were averaged across the school year and used to control for academic achievement. Gender was used to control for the domain-specific development in mathematics and English. Moreover, the academic motivation of the nominated clique members was averaged and used to control for clique-specific norms in mathematics and English.

### Analyses

All models were calculated using MPlus (Muthén and Muthén, 2017). Missing values were estimated with the MLR estimator comparable to the full information maximum likelihood estimator. Moreover, the data were collected within classrooms; thus, the data structure was nested (e.g., Raudenbush and Bryk, 2002). Therefore, the pseudomaximum likelihood estimator (PML, Asparouhov and Muthén, 2005) was used to correct the effects of observation dependencies within classrooms.

To increase the informative value of longitudinal data, True-intradividual-change (TIC) models (Steyer et al., 1997) were calculated. One requirement for calculating change models is time invariance of the constructs (Schmitt et al., 2011). Therefore, the dimensions of Students’ intrinsic value and mastery goals for each subject (mathematics and English) were first tested for their time invariance over three steps (Cheung and Rensvold, 2002). The first step tested the configural invariance. Here, the constructs were modeled following theoretical considerations. The three items of intrinsic value and the four items for mastery goals served as manifest indicators for the latent construct in each subject. All models showed a good fit (see Table 3), indicating configural invariance. The second step, metric invariance over time, was tested by restricting the factor loadings to be equal across time points. The comparison between the configural and metric models showed no systematic difference in all models, indicating metric invariance. In the third step, scalar invariance was tested by fixing the manifest intercepts to be equal between time points in addition to the fixed factor loadings. The results revealed that all motivational variables are scalar or at least partial scalar invariant. Thus, the requirements of invariant constructs across measurement occasions are met. Due to the sample size and the complexity of the calculated True-intradividual-change models, the factor scores of each model were saved and used as indicators. The advantage of factor scores is that they provide partial control for measurement errors in weighting items (Gillet et al., 2017).

**TABLE 3:** Invariance results.

	Configural						Metric			Scalar			Partial scalar		
Model	*X^2^*	*df*	*p*	CFI	RMSEA	SRMR	*Trd*	*df*	*p*	*Trd*	*df*	*p*	*Trd*	*df*	*p*
**Mathematics**															
Intrinsic value	53.00	50	0.38	1.00	0.01	0.02	9.70	8	0.29	13.21	8	0.10			
Mastery goals	130.87	120	0.23	0.99	0.02	0.05	14.66	12	0.26	32.61	12	0.00	6.95	8	0.54
**English**															
Intrinsic value	66.20	50	0.06	0.99	0.03	0.03	5.31	8	0.72	15.70	8	0.047	1.61	4	0.80
Mastery goals	201.35	120	0.00	0.95	0.04	0.06	7.9	12	0.79	12.59	12	0.40			

*Differences in the model fits cannot be determined by conventional χ^2^-difference tests because the fit statistics obtained by the MLR-estimator in Mplus are based on a scaled χ^2^. Therefore, scaled χ^2^ difference test statistics (TRd) were applied (Satorra, 2000; Satorra and Bentler, 2001).*

An intercept for the student T1 value (beginning of the seventh grade) and four change variables across consecutive time points were specified (T1-T2, T2-T3, T3-T4, T4-T5) in the TIC model. These change variables measure the intraindividual changes relative to the baseline. Testing the hypotheses, four models were specified. Each of those models contains a TIC model of one motivational dimension as well as a TIC model of peer group embeddedness as described above. This allowed us to predict changes in the motivational dimensions through changes in peer group embeddedness. In detail, the change variables of peer group embeddedness were regressed on the consecutive change variables of the respective academic motivation dimension controlled for gender, grade and peer group motivation. The effects vice versa were also specified. Testing the second hypothesis, the effects of comparable time points within one school year were tested for significant differences. In the present study, the effects of a change in peer group embeddedness in the first half of the school year on the change in motivational dimensions in the second half of the school year (seventh grade and eighth grade) can be compared. The comparisons were calculated within MPlus using the Model Constraint option.

## Results

### Basic Models

The results of the basic models indicated that on the mean level, most of the changes over the course of the seventh and eighth grades were significantly negative (see Table 4). Especially, in the first half of the year. Moreover, all variances (s^2^) of the changes in intrinsic value and mastery goals were significant. Peer group embeddedness did not change significantly at the mean level. However, similar to the motivational variables, all variances (s^2^) of the changes were significant. Therefore, substantial variations in the change of intrinsic value and mastery goals in mathematics and English can be explained by substantial variations in the change of peer group embeddedness.

**TABLE 4:** Summary statistics (latent level).

		Mathematics		English		
		Intrinsic value	Mastery goal	Intrinsic value	Mastery goal	Embeddedness
**Intercept**						
	Mean	3.09*	3.10*	3.65*	3.51*	0.49*
	Variance	1.04*	0.41*	0.84*	0.44*	0.08*
**Change 1–2**						
	Mean	–0.10**	−0.05*	–0.07	−0.08*	–0.01
	Variance	0.50**	0.18**	0.46**	0.20**	0.12**
**Change 2–3**						
	Mean	0.05	0.04*	−0.05*	0.05	0.01
	Variance	0.33**	0.17**	0.25**	0.20**	0.15**
**Change 3–4**						
	Mean	–0.11**	–0.10**	–0.06	–0.15**	0.02
	Variance	0.33**	0.12**	0.35**	0.16**	0.08**
**Change 4–5**						
	Mean	–0.03	0.02	–0.13**	0.02	–0.02
	Variance	0.34**	0.23**	0.23**	0.15**	0.12**

*p < 0.05, **p < 0.01.

### Effects

**FIGURE 1:**
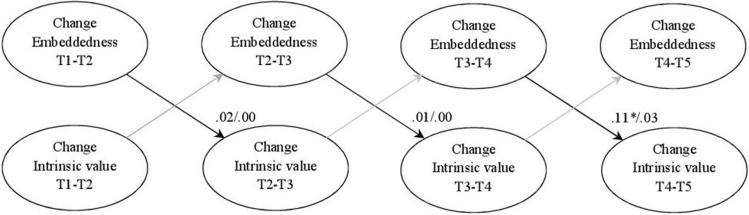
Final true-intraindividual-change model for embeddedness and intrinsic value; coefficients (mathematics/English) represent standardized estimates; *p < 0.05, **p < 0.01.

The direct effects model for intrinsic value in mathematics fit the data well, χ^2^ (20, *n* = 349) = 19.70, *p* = 0.48, RMSEA = 0.00, SRMR = 0.02, CFI = 1.00, TLI = 1.00. The results showed (see Figure 1) that in the first half of seventh grade, a change in peer group embeddedness did not predict a change in Students’ intrinsic value in the second half of the school year. Moreover, a change in peer group embeddedness in the second half of seventh grade also had no effect on the change in Students’ intrinsic value in the first half of the eighth grade. However, in accordance with the hypothesis, a change in peer group embeddedness in the first half of eighth grade significantly predicted a change in Students’ intrinsic value in the second half of the school year. These results indicate that the more Students’ peer group embeddedness increases in the first half of the eighth grade, the more their intrinsic value increases in the second half of the school year. Regarding the subject English, the direct effects model for intrinsic value fit well to the data, χ^2^(20, *n* = 349) = 29.89, *p* = 0.07, RMSEA = 0.04, SRMR = 0.03, CFI = 0.99, TLI = 0.96. Against the hypothesis, a change in peer group embeddedness did not predict any change in Students’ intrinsic value either in seventh grade or in eighth grade.

**FIGURE 2:**
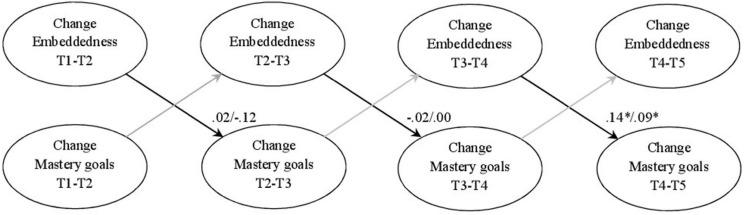
Final true-intraindividual-change model for embeddedness and mastery goals; coefficients (mathematics/English) represent standardized estimates; *p < 0.05, **p < 0.01.

The direct effects model for mastery goals in mathematics also fit the data well, χ^2^(20, *n* = 349) = 20.27, *p* = 0.43, RMSEA = 0.01, SRMR = 0.02, CFI = 1.00, TLI = 1.00. The result pattern was similar to that of Students’ intrinsic value in mathematics (see Figure 2), indicating that only a change in peer group embeddedness in the first half of eighth grade predicted a change in Students’ mastery goals in the second half of eighth grade. The changes in peer group embeddedness in seventh grade did not predict any changes in Students’ intrinsic values. The model for mastery goals in English also fit well to the data, χ^2^(20, *n* = 349) = 11.38, *p* = 0.94, RMSEA = 0.00, SRMR = 0.02, CFI = 1.00, TLI = 1.00. The result pattern was similar to those regarding the motivational variables in mathematics, indicating that only a change in peer group embeddedness in the first half of eighth grade predicted a change in Students’ mastery goals in the second half of eighth grade. The changes in peer group embeddedness in seventh grade did not predict any changes in Students’ mastery goals.

Nearly all effects of the control variables are non-significant, indicating that such short time changes in motivational variables were not influenced through Students’ achievement, gender or group-specific norms.

### Age Effects

In accordance with the second hypothesis, the strength of the effects differed significantly between the beginning of seventh grade and the beginning of eighth grade: intrinsic value mathematics: *T*(1) = 1.91, *p* = 0.03, mastery goals mathematics: *T*(1) = 2.02, *p* = 0.02, and mastery goals English: *T*(1) = 2.55, *p* = 0.01. This indicates that a change in peer group embeddedness is significantly more important for Students’ motivational development in eighth grade than in seventh grade. Due to the non-significant effects of peer group embeddedness on Students’ intrinsic value in English, no further difference test was calculated.

## Discussion

The present study was one of the first to focus on short-term changes in peer group embeddedness and how these changes influence motivational outcomes, such as intrinsic value and mastery goals. A longitudinal study with five measurement occasions makes it possible to determine when a change in peer group embeddedness during early adolescence becomes most important for Students’ development of motivational outcomes. The focus on two domains (mathematics and English) allowed us to validate the results across different subjects.

In line with theoretical explanations (Deci and Ryan, 2000; Ladd et al., 2009), the peer group and the respective change in the level of embeddedness seem to become important resources for promoting student domain-specific intrinsic value and mastery goals, especially in eighth grade. In seventh grade, the results revealed that a change in the level of peer group embeddedness had no effect on the development of all investigated motivational dimensions—nor within the grade level or across the grade levels. This result pattern is also in line within the developmental framework of the present study (e.g., Youniss and Smollar, 1985), that in early adolescence, peer groups become increasingly important for student motivational development with a peak at the age of 14. In contrast to the results of the present study, Wang and Eccles (2012) found that peer support predicted student engagement during adolescence in seventh grade. A possible explanation could be that Wang and Eccles (2012) explicitly focused on friends’ support rather than group-specific constellations and the respective embeddedness, indicating that the influences of dyadic relationships such as friends become important for student motivational development at an earlier developmental stage during early adolescence. Nonetheless, the strength of the effect in both studies is at relatively low. In addition to other aspects in the person and in the environment that may additionally explain student motivational development, it could be assumed that peer group characteristics moderate the effect strength of peer group embeddedness on motivational outcomes (Juvonen, 2006). In this regard, the results of Wang and Eccles (2012) provide the first indications that students who are embedded in an adaptive peer group benefit more from their support than students who are embedded in a maladaptive peer group.

Only the effect pattern for intrinsic value in English differed clearly from all other investigated motivational dimensions. A change in peer group embeddedness had no effect on a change in student intrinsic value in English in either seventh grade or eighth grade. Possible explanations may be derived from student changes in their motivational dimensions in the second half of the eighth grade. The results showed that only Students’ intrinsic value in English declined significantly on the mean level, whereas all other motivational dimensions showed only significant variances in their development. It could only be speculated which external conditions (e.g., curriculum) may be responsible for Students’ unfavorable development of their intrinsic value in English in the second half of the eighth grade. However, the peer group and the respective change in the level of embeddedness seem to act as a protective resource when motivational dimensions change only slightly but not if substantial changes were produced through some external conditions. These *post hoc* interpretations must be investigated more directly in future research.

Moreover, the results revealed that peer group embeddedness—as well as academic motivation—should be understood as a rapidly changing phenomenon in adolescence reflected by considerable changes between the scheduled measurement occasions (4.5 months). These short-term changes might also be more valid in predicting changes in motivational outcomes than one arbitrary measurement occasion, as the non-significant correlations between peer group embeddedness and all motivational dimensions within the time points reflect. In comparison to the development of acceptance (e.g., Jiang and Cillessen, 2005), peer group embeddedness also seems to be less stable over time. Peer group embeddedness indicated by the Bonacich centrality measure is based on the pattering of dyadic interactions rather than general ratings about the likability of a specific person (e.g., acceptance). Thus, it could be assumed that dyadic interactions might be more malleable and dynamic than a general likability of a specific person. Therefore, future studies that focus on peer group embeddedness—assessed via the pattering of dyadic interactions—should consider short-term changes rather than long-term development across several school years (De Laet et al., 2015).

### Contribution and Implications

In sum, the present study contributes to the existing literature in several regards. First, the results revealed that peer group embeddedness indicated by Bonacich network centrality changes to a considerable degree within one school year and even more across school years. Second, the effects showed that in the first half of the eighth grade, the change in peer group embeddedness is most important for the development of motivational dimensions, e.g., mastery goals in mathematics and English and intrinsic value in mathematics. These results extend previous research about different peer relations, such as friends (Molloy et al., 2010; Lessard and Juvonen, 2018) and the whole classroom (Reindl et al., 2015; Wang et al., 2020), and their influences on academic motivation, indicating that each of those relations contributes to the development of motivation.

Implications for future research might be to combine different peer relations, e.g., friends, clique, and the whole classroom community, disentangling the relevance of each relationship for student motivational development. Moreover, the present study investigated a 2-year time period during early adolescence and showed that in the beginning of the eight grades, peer group embeddedness seems to be important for student motivational development. This time period should be extended across the whole of adolescence, disentangling important time points for the importance of peer group embeddedness. Educational practice may also profit from the results. For example, teachers should implement specific interventions for changing and promoting cohesive friendship networks, especially in the beginning of eighth grade, to foster student motivational development.

### Limitations

The present study has several limitations. First, the sample is restricted to the German school system with relatively stable classrooms in the secondary school years. Thus, the results should be replicated in other school systems whose classrooms are more course related. Moreover, the sample is quite small. The results should be replicated with larger samples to test several moderators, such as peer group-specific norms. At least, it has to mention that the non-significant effect of peer group embeddedness in the second half of the seventh grade on the change in motivational outcome in the first half of the eighth grade could be due to the schedule of the measurement occasions. The second measurement occasion took place in the middle of the seventh grade, and the third measurement occasion took place at the beginning of the eight grades after the summer break. During the summer break and irregular meetings with clique members, group constellations may change and in turn influence the level of peer group embeddedness. The non-significant correlations between peer group embeddedness across the two time points provide evidence for this assumption.

## Conclusion

Despite some limitations, the results of the present study suggest that in the first half of eighth grade, a change in peer group embeddedness is important for a change in Students’ academic motivation in the second half of eighth grade. In contrast, a change in peer group embeddedness in seventh grade does not have implications for student motivational development. These grade-specific differences suggest that Students’ sensitivity regarding changes in their peer group embeddedness might increase during early adolescence, which might have consequences for their motivational development in mathematics and English.

## Data Availability Statement

The raw data supporting the conclusions of this article will be made available by the authors, without undue reservation.

## Ethics Statement

Ethical review and approval was not required for the study on human participants in accordance with the local legislation and institutional requirements. Written informed consent to participate in this study was provided by the participants’ legal guardian/next of kin.

## Author Contributions

MR was responsible for the conception and the design of the work, the acquisition, analysis, and interpretation of data for the work.

## Conflict of Interest

The author declares that the research was conducted in the absence of any commercial or financial relationships that could be construed as a potential conflict of interest.

## Publisher’s Note

All claims expressed in this article are solely those of the authors and do not necessarily represent those of their affiliated organizations, or those of the publisher, the editors and the reviewers. Any product that may be evaluated in this article, or claim that may be made by its manufacturer, is not guaranteed or endorsed by the publisher.
